# Incidence and Characteristics of Retinoblastoma in Poland: The First Nationwide Study 2010–2017

**DOI:** 10.3390/ijerph18126539

**Published:** 2021-06-17

**Authors:** Michał S. Nowak, Bożena Romanowska-Dixon, Iwona Grabska-Liberek, Michał Żurek

**Affiliations:** 1 Provisus Eye Clinic, 112 Redzinska str., 42-209 Czestochowa, Poland; 2 Saint Family Hospital Medical Center, 19 Wigury Str., 90-302 Lodz, Poland; 3 Department of Ophthalmology and Ophthalmic Oncology, Jagiellonian University Collegium Medicum, 31-501 Cracow, Poland; romanowskadixonbozena1@gmail.com; 4 Department of Ophthalmology, Centre of Postgraduate Medical Education, 01-416 Warsaw, Poland; iliberek@gmail.com; 5 Department of Analyses and Strategies, Ministry of Health, 00-952 Warsaw, Poland; m.zurek@mz.gov.pl; 6 Doctoral School, Medical University of Warsaw, 02-091 Warsaw, Poland

**Keywords:** retinoblastoma, eye surgery, chemotherapy, radiotherapy

## Abstract

*Background*: The present study aimed to investigate the incidence and characteristics of retinoblastoma in the overall population of Poland. *Methods*: The retrospective survey of both National Health Fund (NHF) and National Cancer Registry (NCR) databases were performed to identify all retinoblastoma cases in Poland in the years 2010–2017. *Results*: During 2010–2017, the mean age-standardised incidence of retinoblastoma (the unit of incidence is per 1,000,000 person-years) was 10.15 (95% CI 7.23–13.08) among children aged 0 to 4 years and 5.39 (95% CI 4.18–6.60) in those aged 0 to 9 years. During 2010–2014 (to allow 5 years of follow-up), the mean incidence of retinoblastoma by birth cohort analysis in Poland was 4.89 (95% CI 4.04–5.74) per 100,000 live births, corresponding to an incidence of 1 per 20,561 (95% CI 15,855–25,267) live births. In Poland, 14.6% of children with retinoblastoma had enucleation of the eye globe, 76.8% received different types of chemotherapy combined with focal treatment, 5.9% were treated with external beam radiotherapy, and 2.7% were treated with focal treatments only. *Conclusions*: The incidence of retinoblastoma and the pattern of medical management of retinoblastoma in Poland was similar to that reported in developed countries in Western Europe, Asia, and North America.

## 1. Introduction

Retinoblastoma is the most common intraocular paediatric eye cancer. It accounts for approximately 11% of primary cancers occurring in the first year of life, with 95% of diagnoses before 5 years of age [1,2,3]. Since retinoblastoma is very aggressive, early diagnosis and prompt treatment are vital for children to preserve their sight and life. In western countries in Europe and North America, retinoblastoma is diagnosed early so the chances of saving the patient’s life and preserving vision are good. However, in large regions of Africa and Asia, where the diagnosis of retinoblastoma is delayed, the deadly effect is observed [2,3,4,5]. In these regions, socioeconomic factors and poor recognition of the seriousness of the disease result in a high mortality rate of up to 70%. At the same time, the mortality rate in Europe and North America is 3–5% [2]. Besides, the incidence of retinoblastoma also varies by race and region, from 40 to 60 per million live births, which corresponds to 1 per 16,000–24,000 live births, with the greatest disease burden recorded in the countries of Asia and Africa where the highest birth rates are recorded [2,3]. However, there is a lack of data from Eastern European countries like Poland [1,3,4,5,6,7,8,9,10,11].

The management of retinoblastoma may also differ between developed and developing countries. The current strategy of retinoblastoma treatment aims to save the life, eye, vision, and cosmetics of the child, in order of priority. Medical management of retinoblastoma includes surgical treatment (enucleation), external beam radiotherapy, systemic chemotherapy with or without focal therapies such as cryotherapy, laser photocoagulation, and plaque radiotherapy. Recently, intra-arterial and intravitreal chemotherapies have been used to treat retinoblastoma with the ability to save globes that otherwise would have been enucleated [2,3].

The present study aimed to investigate the incidence and characteristics of retinoblastoma in the overall population of Poland in the years 2010–2017 and to report the changes that occurred during that period.

## 2. Materials and Methods

### 2.1. Data Sources, Disease Codes, and Definitions

The present study was a part of the Polish Ministry of Health project “Maps of Healthcare Needs—Database of Systemic and Implementation Analyses” and was co-financed by the European Union funds through the European Social Fund under the Operational Program of Knowledge Education and Development (EU grant number: POWR 05.02.00-00-0149/15-01) [12,13,14]. The study design was a retrospective and nationwide survey. The data concerning all patients who were diagnosed with retinoblastoma in Poland between January 2010 and December 2017, obtained from the National Health Fund (NHF) database of medical service, were assessed and matched with the National Cancer Registry (NCR) [15,16]. In Poland, information related to all levels of healthcare services at public and private institutions financed from public sources is recorded in the NHF database. The information includes medical data and demographical features like personal identification number (PESEL), date of birth, area code, and sex of patients. The medical data include the diagnoses coded according to the International Classification of Diseases, 10th Revision (ICD-10), and all performed procedures coded using the International Classification of Diseases, 9th Revision (ICD-9), procedure codes and unique NHF codes corresponding to certain hospital procedures. All patients with confirmed cancer diagnoses are also registered in the National Cancer Registry (NCR). As the NCR uses the NHF database covering the entire population of Poland, it is believed to ascertain all retinoblastoma cases diagnosed in Poland. During the study period, in the years 2010–2017, each child reported in both NHF and NCR databases with a confirmed primary diagnosis of retinoblastoma was retrospectively identified with ICD-10 code C69.2. The ICD-9 codes 16.39, 16.42, and 16.49 were used to identify retinoblastoma treatment by surgery. Chemotherapy treatment was identified with ICD-9 code 99.25 and external beam radiotherapy was identified with ICD-9 codes 99.29 and 14.26 and 14.27. Focal treatment methods were identified with the following ICD-9 codes: 99.27 and 14.27 for plaque radiotherapy, 14.24 and 14.25 for laser therapy, and 14.22 and 14.32 for cryotherapy. The population data for Poland were obtained from Statistics Poland [17].

In the first part of the study, the descriptive statistics of the retinoblastoma incidence in Poland during 2010–2017 were performed. The analysis included the incidence of retinoblastoma per live birth by birth cohort analysis during 2010–2014 estimated as the number of children who developed retinoblastoma among those born in each year (birth cohorts) divided by the total number of live-born children in that year. This allowed 5 years of follow-up for the last analysed birth cohort and 95% confidence interval (CI) of the incidence rate was estimated based on the normal distribution. Other analyses included the age-standardised incidence of retinoblastoma (the unit of incidence is per 1,000,000 person-years) during the entire study period. The incidence of retinoblastoma was presented for each year separately, by age category matched with corresponding year population data in Poland, and was calculated by dividing the number of children diagnosed with retinoblastoma by the number of children in the same age group or by the total number of children in the studied year. Other statistical analyses included demographic characteristics of children with retinoblastoma (the socio-demographic data, including age, sex, and place of residence, were anonymously recorded) and retinoblastoma treatment characteristics by treatment procedure surgery, chemotherapy, radiotherapy, and focal therapies. We also performed the statistical analysis of the trend in the number of procedures in the analyzed period. The analyzed period was divided into two ranges—from 2010 to 2013 and from 2014 to 2017. The proportions of procedures in both ranges were calculated and compared using two-proportions Z-Test. *p* values < 0.05 were considered statistically significant.

### 2.2. Survival Analysis

The second part of the present study focused on survival analysis conducted in all patients diagnosed with retinoblastoma during the entire study period using the logistic regression model. The Kaplan-Meier curve was used to present the patient survival. To estimate the survival rate of retinoblastoma patients, the NCR matched the cases diagnosed during 2010–2017 to the vital status of the patient using information obtained from Statistics Poland [17]. The odds ratios (ORs) were used to verify the risk factors (*p* values < 0.05 were considered statistically significant). R statistical software V. 3.6.2 (R Foundation for Statistical Computing, Vienna, Austria) was used for all analyses.

The study adhered to the tenets of the Declaration of Helsinki for research involving human subjects (socio-demographic data, including age, sex, and place of residence, were anonymously recorded) but we did not need any ethic committee approval. The study protocol was approved by the Polish Ministry of Health, which is entitled by the law of the Republic of Poland to process the National Health Fund data.

## 3. Results

**Figure 1:**
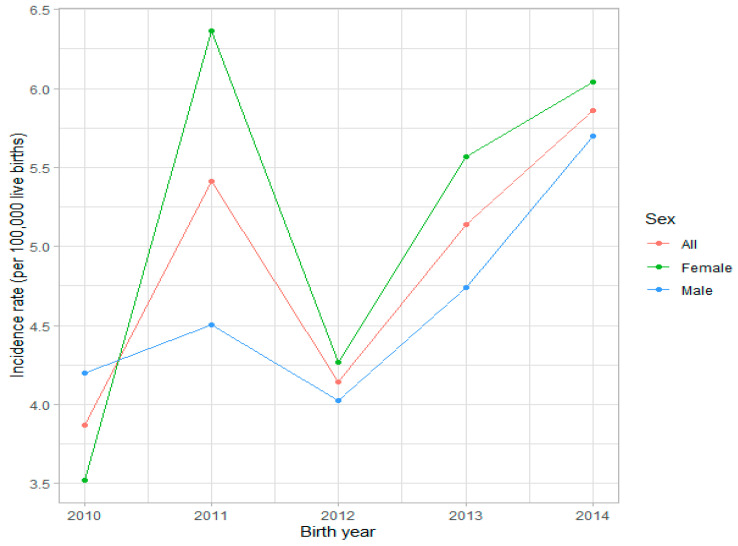
Incidence of retinoblastoma in Poland per live birth by birth cohort analysis during 2010–2014.

**Figure 2:**
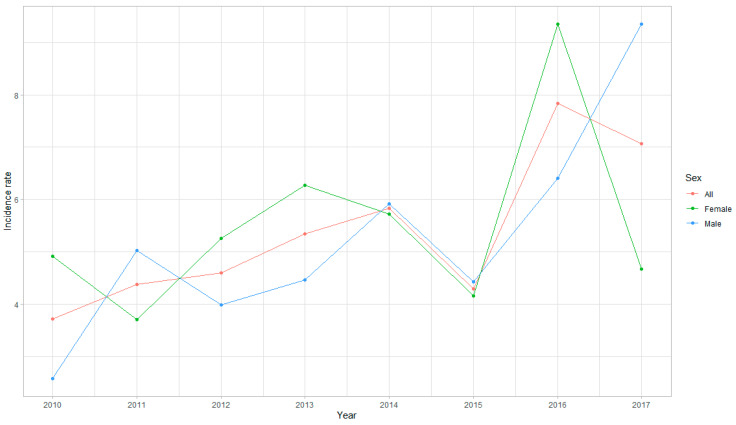
Incidence of retinoblastoma in Polish children by standard annual analysis during 2010–2017.

The incidence of retinoblastoma in Poland by birth cohort and standard annual analyses are presented in Figure 1 and Figure 2, Table 1 and Table 2. During the study period, the total number of 185 children with retinoblastoma were identified in both NHF and NCR registers in Poland. Based on the aforementioned criteria, 169 of those children were born alive between 2010 and 2017. The other 16 of children with retinoblastoma—who were born alive before 2010—were included in the retinoblastoma treatment analysis only. We did not find any child with retinoblastoma aged ≥ 10 years at the time of diagnosis. The majority of cases appeared before 5 years of age, and only 10 out of 169 children (5.9%) were 5 years or older at the time of diagnosis in Poland (Table 2). The mean incidence of retinoblastoma by birth cohort analysis in Poland was 4.89 (95% CI 4.04–5.74) per 100,000 live births during the period 2010–2014, corresponding to an incidence of 1 per 20,561 (95% CI 15,855–25,267) live births. Detailed incidence rate per live birth by birth cohort analysis in each year during the period 2010–2014 is presented in Figure 1 and Table 1. During the period 2010–2017, the mean age-standardised incidence of retinoblastoma (the unit of incidence is per 1,000,000 person-years) was 10.15 (95% CI 7.23–13.08) among children aged 0 to 4 years and 5.39 (95% CI 4.18–6.60) in those aged 0 to 9 years. The detailed incidence rate of retinoblastoma in Poland by standard annual analysis during the period 2010–2017 is presented in Figure 2 and Table 2.

**Figure 3:**
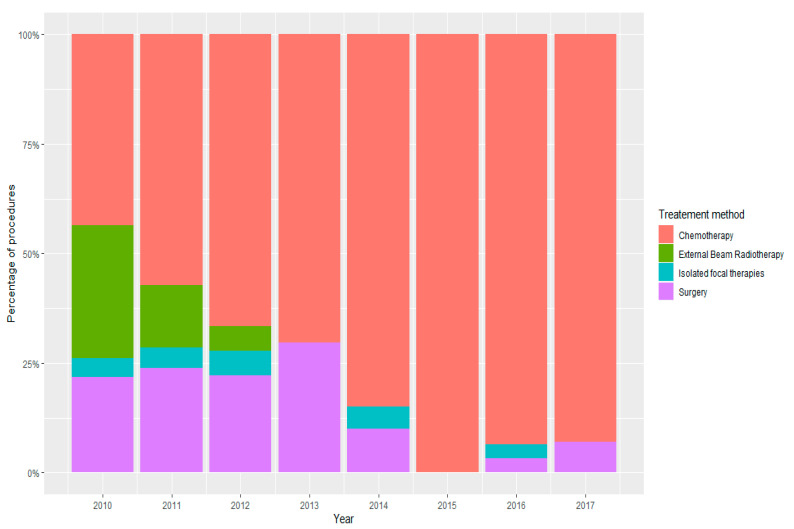
Characteristic of retinoblastoma treatment methods in Polish children during 2010–2017.

Demographic and clinical characteristics of children with retinoblastoma in Poland in the years 2010–2017 are presented in Figure 3, Table 3 and Table 4. In the study period, the mean age at the time of diagnosis was 1.63 ± 1.66 years. Females represented 49.7% of all children registered with retinoblastoma. In Poland, 68.6% of retinoblastoma patients in the years 2020–2017 lived or had lived in urban areas. During the analysed period, 14.6% of children had surgery (enucleation of the eye globe), 76.8% received different types of chemotherapy combined with focal treatment, 5.9% were treated with external beam radiotherapy, and 2.7% were treated with focal methods only, including plaque radiotherapy, laser photocoagulation of the retina, and cryotherapy. However, different treatment patterns were observed between age groups. Among children aged 0 to 4 years, 13.9% of children had enucleation, 77.4% received different types of chemotherapy combined with focal treatment, 5.8% were treated with external beam radiotherapy, and 2.9% were treated with focal methods only. However, in children aged 5 to 9 years, 25% of children had enucleation, 67.7% received different types of chemotherapy combined with focal treatment, 8.3% were treated with external beam radiotherapy, and none were treated with isolated focal methods. The time trends in the medical management of retinoblastoma patients were also observed in the analysed period in Poland. The total number of children who had chemotherapy combined with focal treatment increased from 43.5% in 2010 to 93.1% in 2017, the number of enucleations decreased from 21.7% in 2010 to 6.9% in 2017, and the number of external beam radiotherapies and isolated focal therapies decreased from 30.4% and 4.4% in 2010 to 0.0% and 0.0% in 2017, respectively. The increase in chemotherapy combined with focal treatment and the decreases of enucleations and external beam radiotherapies were statistically significant in the study period (*p* = 0.00002, *p* = 0.00001 and *p* = 0.00002, respectively).

### Survival Rate and Logistic Regression Analysis

**Figure 4:**
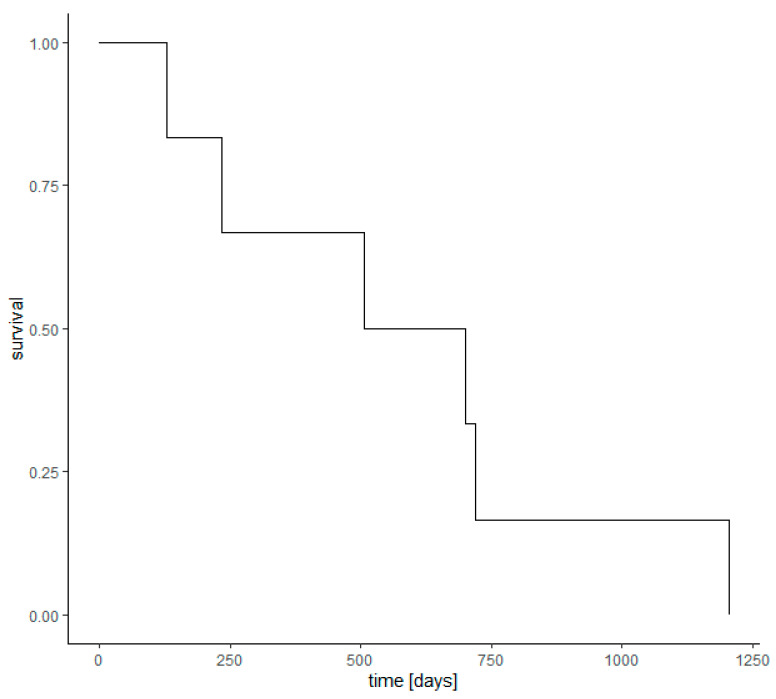
Kaplan-Meier curve of retinoblastoma survival in Poland.

Survival analysis was conducted on all 169 children diagnosed with retinoblastoma between 2010 and 2017. The Kaplan-Meier curve (Figure 4) presents the patient survival. In Poland, only 6 children born alive during the study period have died from retinoblastoma, which gives a mortality rate of 3.55%. The logistic regression analysis showed that children aged 0–4 years at the time of diagnosis had a significantly lower risk of death with OR 0.075 (*p* = 0.009). However, sex and residence of children were not associated with the mortality rate. The medical management of retinoblastoma was not significantly associated with patient survival either (Table 5).

## 4. Discussion

This study evaluates the incidence and characteristics of retinoblastoma in Poland in the years 2010–2017. Since the study is country-based and covers the overall population of Poland, it provides for the first time data on retinoblastoma for the Eastern European region. The analysis included both the incidence of retinoblastoma per live birth by birth cohort analysis during 2010–2014 and the age-standardised incidence of retinoblastoma (the unit of incidence is per 1,000,000 person-years) during the entire study period. The incidence per live birth is a traditional method for calculating the retinoblastoma incidence relative to live births in a given year. The age-standardised incidence of retinoblastoma is most commonly calculated for children aged under 5 years because the incidence in children aged over 5 years is very low and is estimated to be no more than 0.3 to 0.5 per 1 million [4]. The mean incidence of retinoblastoma by birth cohort analysis in Poland was 4.89 per 100,000 live births during the period 2010–2014, corresponding to an incidence of 1 per 20,561 live births. Our results were in the middle of the range of retinoblastoma incidence by birth cohort worldwide [2,3]. In Poland, during the period 2010–2017, the mean age-standardised incidence of retinoblastoma was 10.2 per 1,000,000 person-years among children aged 0 to 4 years and 5.4 in those aged 0 to 9 years, with the mean age of 1.63 ± 1.66 years at the time of diagnosis. The age-standardised incidence of retinoblastoma shown here was similar to that reported in the previous study from Great Britain and lower than that reported in South Korea, Singapore, the USA, and in Northern Europe (Sweden and Finland). A comparison of the reported annual incidence of retinoblastoma in nationwide populations against the previously published studies is presented in Table 6. The retinoblastoma incidence rate was similar between the sexes in Poland, which is in agreement with most of the other studies. In contrast, the studies from the USA and Taiwan showed a higher incidence rate of retinoblastoma among boys compared with girls [1,3].

Another important part of our study was the analysis of the medical management of retinoblastoma in Poland. However, since the 1990s, chemotherapy has been widely used as a primary treatment for retinoblastoma to reduce tumour size before focal therapies, the only definitive cure for retinoblastoma (which saves life) is the removal of the eye globe before the tumour spreads [2,18]. The secondary aim to save vision can be achieved with expensive treatment methods, which are not available in most countries of low and middle income. Current globe-sparing therapies, including periocular carboplatin, selective ophthalmic artery chemoreduction, and intravitreal melphalan combined with focal therapies, are being used and investigated actively [19]. In Poland, the total number of children undergoing chemotherapy combined with focal treatment increased significantly from 2010 to 2017. In the same period, the number of enucleations, external beam radiotherapies, and isolated focal therapies decreased. These time trends in the medical management of retinoblastoma patients observed in Poland were in agreement with the trends of retinoblastoma management observed in the United States of America where the use of external beam radiotherapy to treat retinoblastoma has decreased from 30% of treatments in the period from 1973 to 1976 to 2% in the period from 2005 to 2008 [20]. However, different treatment patterns were observed between age groups in Poland, with a higher rate of enucleations in the age group of 5–9 years.

The survival analysis revealed that only six children born alive in Poland during the study period died from retinoblastoma, which gives a mortality rate of 3.55%. Although Poland is a middle-income country [21], its mortality rate was comparable to that found in developed countries. It was lower than Singapore, South Korea, and Japan but higher than Great Britain [8,9,11,22]. The logistic regression model with age, sex, place of residence, and medical management of retinoblastoma patients was constructed to analyze the risk factors of mortality. Our analysis showed that children aged 0–4 years at the time of diagnosis had 13.3 times lower risk of death compared to the age group of 5–9 years (OR = 0.075). However, sex, place of residence of children, and medical management of retinoblastoma were not statistically associated with the mortality rate. The results of the study from Taiwan showed that the surgical intervention of enucleation reduced mortality rate 3.7 times (OR = 0.27) when compared with other treatment methods [3].

There are some limitations to the present study. First, both NHF and NCR databases do not include family history, pathologic reports, and genetic information of retinoblastoma patients in Poland. Second, the cancer stage and laterality are also not available for all included subjects. Therefore, we were unable to recognize the familial cases and the investigation of potential risk factors of mortality was not complex. However, this likely had only a minor impact on the study findings. The population size, national recruitment, and impact of its findings on public health care policy are the most important strengths of the present study. However, our results are specific to Poland and cannot describe other Eastern European populations.

## 5. Conclusions

This is the first nationwide report of the retinoblastoma epidemiology in Poland. To the best of our knowledge, there are no published national epidemiological studies of retinoblastoma from Eastern Europe. This study shows that the incidence of retinoblastoma, the mortality rate of patients with retinoblastoma, and the pattern of medical management of retinoblastoma in Poland was similar to that reported in developed countries in Western Europe, Asia, and North America. This retrospective epidemiological study on retinoblastoma will help clinicians and healthcare institutions to better manage and treat retinoblastoma patients in Poland.

## Figures and Tables

**Table 1:** Incidence of retinoblastoma in Poland per live birth by birth cohort analysis during 2010–2014.

	2010	2011	2012	2013	2014	All
No. of live births	413,425	388,468	386,104	369,558	375,166	1,932,721
No. of retinoblastoma	16	21	16	19	22	94
Incidence per 100,000 live births	3.87	5.41	4.14	5.14	5.86	4.86
No. of live births per 1 retinoblastoma	1:25,839	1:18,499	1:24,132	1:19,450	1:17,053	1:20,561

Mean incidence with 95% confidence interval: 4.89 ± 0.85.

**Table 2:** Incidence of retinoblastoma in Polish children by standard annual analysis during 2010–2017.

	2010	2011	2012	2013	2014	2015	2016	2017
No. age 0–4 years (in thousands)	1981.4	2066.6	2064.9	2023.1	1962.1	1911.3	1882.1	1888.8
No. of retinoblastoma	11	16	17	20	22	16	30	27
Incidence/1,000,000 person-yrs	5.55	7.74	8.23	9.89	11.21	8.37	15.94	14.29
No. age 5–9 years (in thousands)	1783.3	1811.3	1843.4	1907	1984.1	2044	2072.4	2072.3
No. of retinoblastoma	3	1	1	1	1	1	1	1
Incidence/1,000,000 person-yrs	1.68	0.55	0.54	0.52	0.50	0.49	0.48	0.48
All children (in thousands)	3764.7	3877.9	3908.3	3930.1	3946.2	3955.3	3954.5	3961.1
No. of retinoblastoma	14	17	18	21	23	17	31	28
Incidence/1,000,000 person-yrs	3.72	4.38	4.61	5.34	5.83	4.30	7.84	7.07

Incidence 10.15/1,000,000 person-yrs < 5 years of age; Incidence 5.39/1,000,000 person-yrs < 10 years of age.

**Table 3:** Demographic characteristics of Polish children with retinoblastoma during 2010–2017.

	2010	2011	2012	2013	2014	2015	2016	2017	All
Age mean ± SD	2.71 ± 2.89	1.53 ± 1.62	2.11 ± 1.81	1.38 ± 1.36	1.35 ± 1.4	1.41 ± 1.42	1.32 ± 1.14	1.71 ± 1.67	1.63 ± 1.66
Female (%)	64.29%	41.18%	55.56%	57.14%	47.83%	47.06%	58.06%	32.14%	49.70%
Male (%)	35.71%	58.82%	44.44%	42.86%	52.17%	52.94%	41.94%	67.86%	50.30%
Urban residence (%)	78.57%	58.82%	66.67%	76.19%	78.26%	64.71%	74.19%	53.57%	68.64%
Rural Residence (%)	21.43%	41.18%	33.33%	23.81%	21.74%	35.29%	25.81%	46.43%	31.36%

**Table 4:** Characteristic of retinoblastoma treatment methods in Polish children during 2010–2017.

Year	Surgery Enucleation *n*;%	Chemotherapy with Focal Therapies *n*;%	External Beam Radiotherapy *n*;%	Isolated Focal Therapies *n*;%	All *n*;%
0–4 years					
2010	4 (21.05%)	8 (42.11%)	6 (31.58%)	1 (5.26%)	19 (100%)
2011	4 (21.05%)	11 (57.89%)	3 (15.79%)	1 (5.26%)	19 (100%)
2012	4 (23.53%)	11 (64.71%)	1 (5.88%)	1 (5.88%)	17 (100%)
2013	7 (28%)	18 (72%)	0 (0%)	0 (0%)	25 (100%)
2014	2 (10%)	17 (85%)	0 (0%)	1 (5%)	20 (100%)
2015	0 (0%)	15 (100%)	0 (0%)	0 (0%)	15 (100%)
2016	1 (3.33%)	28 (93.33%)	0 (0%)	1 (3.33%)	30 (100%)
2017	2 (7.14%)	26 (92.86%)	0 (0%)	0 (0%)	28 (100%)
Total	24 (13.87%)	134 (77.46%)	10 (5.78%)	5 (2.89%)	173 (100%)
5–9 years					
2010	1 (25%)	2 (50%)	1 (25%)	0 (0%)	4 (100%)
2011	1 (50%)	1 (50%)	0 (0%)	0 (0%)	2 (100%)
2012	0 (0%)	1 (100%)	0 (0%)	0 (0%)	1 (100%)
2013	1 (50%)	1 (50%)	0 (0%)	0 (0%)	2 (100%)
2014	0 (0%)	0 (0%)	0 (0%)	0 (0%)	0 (100%)
2015	0 (0%)	1 (100%)	0 (0%)	0 (0%)	1 (100%)
2016	0 (0%)	1 (100%)	0 (0%)	0 (0%)	1 (100%)
2017	0 (0%)	1 (100%)	0 (0%)	0 (0%)	1 (100%)
Total	3 (25%)	8 (66.67%)	1 (8.33%)	0 (0%)	12 (100%)
All children					
Year	Surgery Enucleation *n*;%	Chemotherapy with focal therapies *n*;%	External Beam Radiotherapy *n*;%	Isolated focal therapies *n*;%	All *n*;%
2010	5 (21.74%)	10 (43.48%)	7 (30.43%)	1 (4.35%)	23 (100%)
2011	5 (23.81%)	12 (57.14%)	3 (14.29%)	1 (4.76%)	21 (100%)
2012	4 (22.22%)	12 (66.67%)	1 (5.56%)	1 (5.56%)	18 (100%)
2013	8 (29.63%)	19 (70.37%)	0 (0%)	0 (0%)	27 (100%)
2014	2 (10%)	17 (85%)	0 (0%)	1 (5%)	20 (100%)
2015	0 (0%)	16 (100%)	0 (0%)	0 (0%)	16 (100%)
2016	1 (3.23%)	29 (93.55%)	0 (0%)	1 (3.23%)	31 (100%)
2017	2 (6.9%)	27 (93.1%)	0 (0%)	0 (0%)	29 (100%)
Total	27 (14.59%)	142 (76.76%)	11 (5.95%)	5 (2.7%)	185 (100%)

Z-test; *p* = 0.00001; *p* = 0.00002; *p* = 0.00002; *p* = 0.206.

**Table 5:** Results of the logistic regression model of survival analysis conducted in all patients diagnosed with retinoblastoma during 2013–2017 in Poland.

Variable	Β	SE	*p* Value	OR	2.5% OR	97.5% OR
Intercept	−0.492	1.506	0.744	0.61145309	0.01916875	9.8735799
rural_residence	−0.045	0.957	0.963	0.95635294	0.11166805	5.7354009
sex_male	−0.185	0.884	0.835	0.83147841	0.13349941	5.0228598
age_0–4	−2.591	0.992	0.009	0.07497658	0.01083035	0.6403799
retinoblastoma treatment with surgery-enucleation	−17.09	1926.242	0.993	0.00000004	0	4.89 × 10^59^
retinoblastoma treatment without surgery	−0.353	1.191	0.767	0.70236958	0.09024939	15.1361955

**Table 6:** Comparison of reported annual incidence of retinoblastoma in nationwide populations from previously published studies.

Epidemiological Study	Study Period	Age < 5 Years Per Million	Age < 10 Years Per Million	Live Births
Sweden [4]	1958–1998	11.8	NA *	1:14,900
Finland [4]	1958–1988	11.2	NA *	1:16,100
Finland [5]	1964–2014	12.1	6.5	1:16,130
Netherlands [6]	1862–1995	NA *	NA *	1:17,000
Singapore [7,8]	1968–1995	11.1	2.4	NA *
Great Britain [9]	1963–2002	10.0	3.5	NA *
USA [10]	1975–2004	11.8	NA *	NA *
USA [1]	2000–2009	from 11.2 to 13.2 **	NA *	NA *
South Korea [11]	1993–2020	11.2	5.3	1:16,900
Taiwan [3]	1998–2011	NA *	NA *	1:17,373
Poland (present study)	2010–2019	10.2	5.4	1:20,561 ***

* Data not available; ** 11.2 in males and 13.2 in females; *** birth cohort analysis during 2010–2014.

## Data Availability

The National Health Fund Registry data are available at http://www.nfz.gov.pl (accessed on 1 December 2020) and the Statistics Poland data are available at http://www.stat.gov.pl (accessed on 1 December 2020).
